# Real-world TRAE association between niraparib and platinum-based chemotherapy

**DOI:** 10.3389/fonc.2024.1390820

**Published:** 2024-06-17

**Authors:** Linli Wang, Jieli Zhou, Haibin Wang, Wenling Han, Chunyun Fang

**Affiliations:** ^1^ First Clinical College, Gannan Medical University, Ganzhou, China; ^2^ Department of Obstetrics and Gynecology, First Affiliated Hospital of Gannan Medical University, Ganzhou, China

**Keywords:** ovarian cancer, niraparib, chemotherapy, PARPi, platinum drugs, hematologic adverse reactions

## Abstract

**Background:**

Pre-clinical studies showed the anti-tumor mechanisms of PARP inhibitors (PARPi) and platinum have some crossover and overlap in the DNA damage repair pathway, patients who respond to platinum-based chemotherapy are also more likely to be sensitive to PARPi. This real-world study mainly aimed to evaluate whether TRAE (treatment-related adverse event) between platinum based chemotherapy (PBC) and niraparib are also associated.

**Methods:**

Patients received niraparib as maintenance treatment or salvage therapy for advanced ovarian cancer at the First Affiliated Hospital of Gannan Medical University from January 2020 to August 2023 were included. Survival data of niraparib treatment and adverse events occurred during the last platinum-based chemotherapy cycle before starting niraparib treatment and during niraparib treatment are documented. Fisher’s exact test were used for correlation analysis.

**Results:**

1. 40 patients treated with niraparib were included in the analysis, including 31 patients treated with niraparib for 1st-line maintenance therapy, 6 patients for PSR (platinum-sensitive recurrence) maintenance therapy, and 3 patients for salvage therapy. The overall median follow-up time was 15.0 months (ranged from 2.2 months to 32.1 months). 2. Overall grade≥3 TRAE (40% vs 70%, p=0.012) including anemia (20% vs 45%, p=0.041) and neutrophil count decreased (17.5% vs 57.5%, p<0.001) was significantly lower during niraparib treatment compared to during chemotherapy. 3. Any grade TRAE (75% vs 100%, p=0.002) including white blood cell count decreased (47.5% vs 87.5%, p<0.001), red blood cell count decreased (57.5% vs 92.5%, p<0.001), anemia (55% vs 87.5%, p<0.001) and neutrophil count decreased (35% vs 85%, p<0.001) were also significantly lower in niraparib treatment group compared with chemotherapy group. No new safety signals were identified.

**Conclusion:**

1. In this real-world practice, we observed that patients with advanced ovarian cancer who experienced any grade and grade ≥3 TRAE during chemotherapy were well tolerated when treated with niraparib, particularly the incidence of any grade and grade ≥3 anemia, and neutrophil count decreased during niraparib treatment were significantly lower compared with that during chemotherapy. 2. For patients with ovarian cancer who have experienced grade ≥3 hematological adverse reactions during prior platinum-based chemotherapy, greater attention should be paid to the monitoring and management of hematological adverse reactions during subsequent treatment with niraparib.

## Background

Ovarian cancer is the eighth most common cancer among females. 90% of ovarian cancers are of an epithelial cell type and comprise multiple histologic types, with various specific molecular changes, clinical behaviors, and treatment outcomes. The remaining 10% are non-epithelial ovarian cancers, which include mainly germ cell tumors, sex cord-stromal tumors, and some extremely rare tumors such as small cell carcinomas. Germ cell tumors are the most common ovarian neoplasms in women until 30 years of age and most of the patients are diagnosed with early-stage disease (60–70%) (1). In 2020, 313,959 women worldwide were newly diagnosed with ovarian cancer, and 207,252 women died from the disease (2). The incidence of ovarian cancer in China is increasing and ranks third among malignant tumors of the female reproductive system, with the highest mortality rate. Currently, there is no effective early screening strategy for ovarian cancer, and the early symptoms are often hidden (3). This is also reflected economically and cost-effective strategies for early detection and prevention of ovarian cancer have been investigated over the last decade. The cost of treatment per patient with ovarian cancer remains the highest among all cancer types. As an example, the average initial cost in the first year can amount to around USD 80,000, whereas the final year cost may increase to USD 100,000 (4). Type I epithelial ovarian cancers are suggested to be relatively indolent and genetically stable tumors that typically arise from recognizable precursor lesions, such as endometriosis or borderline tumors with low malignant potential. In contrast, type II epithelial ovarian cancers are proposed to be biologically aggressive tumors from their outset, with a propensity for metastasis from small-volume primary lesions. High-grade serous – the most common type of epithelial ovarian cancers, accounting for approximately 75% of epithelial ovarian cancers – develop according to the type II pathway and present p53 and BRCA mutations (5).the p53 genes involved in DNA repair, the cell cycle, and apoptosis upon irreparable DNA damage (6). The DNA double-strand breaks are repaired by the homologous recombination repair pathway, which is an error-free process requiring a homologous DNA template to function (6). BRCA1, BRCA2, and various other homologous recombination proteins are responsible for the repair of DNA damage that maintains genomic stability and promotes cell survival and replication. Ovarian cancers with BRCA1 alterations (germline and somatic mutations in 12% of cases, DNA hypermethylation in 11% of cases) and BRCA2 alterations (germline and somatic mutations in 11% of cases) (7), are associated with homologous recombination deficiency (HRD). The finding that HRD contributes to approximately 50% of HGSOCs provided a rationale for using cytotoxic platinum-based chemotherapy and exploring the activity of poly (ADPribose) polymerase (PARP) inhibitors in HGSOC (7).Approximately 70% of ovarian cancer patients are diagnosed at an advanced stage, and about 80% of those with advanced stage experience recurrence within 3 years after chemotherapy remission. As the number of treatment lines increases, the platinum-free interval becomes shorter, ultimately leading to platinum resistance. The 5-year survival rate is only 15%-25% (6, 8). As proteomics continues to be studied, such as mass spectrometry and protein array analysis, which have advanced the dissection of the underlying molecular signaling events and the proteomic characterization of ovarian cancer (9). While the over or under expression of certain proteins may indicate reduced sensitivity to chemotherapy, emerging evidence shows that targeted treatment against the pathways conferring resistance may help to overcome it (10).The Cancer Genome Atlas and the International Cancer Genome Consortium have sequenced thousands of ovarian tumor specimens, which has resulted in the identification of novel genomic sequences that could be targets for therapeutic interventions (11), poly (ADP-ribose) polymerase (PARP) inhibitors are one of the two drugs with the best evidence for FDA approval for the treatment of ovarian cancer (12).In recent years, targeted therapy research has advanced, shifting the treatment approach for epithelial ovarian cancer from the traditional ‘tumor cytoreductive surgery + platinum-based chemotherapy’ mode to a ‘tumor cytoreductive surgery + platinum-based chemotherapy + long-term disease management mode of maintenance therapy’. PARP inhibitors have emerged as an important means of maintaining ovarian cancer. However, there is currently a lack of data on the correlation between real-world niraparib use and hematologic adverse reactions (TRAE) that occur during platinum-based chemotherapy in ovarian cancer patients. Therefore, our study aims to analyze the real-world association between niraparib and TRAE during platinum-based chemotherapy.

## Methods

### Study population

Patients with ovarian cancer who received platinum-based chemotherapy and niraparib successively in the First Affiliated Hospital of Gannan Medical University from January 2020 to May 2023 were enrolled. Including patients with newly treated advanced epithelial ovarian cancer who achieved complete response(CR) or partial response(PR) after platinum-based chemotherapy, patients with platinum-sensitive recurrent ovarian cancer who achieved CR or PR after platinum-based chemotherapy, ovarian cancer achieves CR or PR or stable disease(SD) to multiple lines(≥2 lines and platinum resistance)of platinum-based chemotherapy. Follow-up ended on August 31, 2023. Baseline data of the patients were collected, including the patient’s age, weight, family history, clinical stage of the International Federation of Obstetrics and Gynecology (FIGO), pathological type, presence of other comorbidities before chemotherapy, Eastern Cooperative Oncology Group (ECOG) score, Frontline platinum-based chemotherapy cycles,number of front-line chemotherapy lines, The last line of chemotherapy regimen prior to treatment with niraparib. The hematologic adverse reactions during chemotherapy, ≥ grade 3 adverse reactions during chemotherapy, the end time of the last platinum-containing chemotherapy, and the response of previous first-line platinum-based chemotherapy based on RECIST1.1 assessment. Additionally, the baseline number of platelets and CA125 before niraparib treatment, BRCA mutation status, adjuvant therapy, and follow-up after the use of niraparib were documented.

### Group standard

Inclusion Criteria: (1) Patients aged ≥18 years and signed an informed consent form related to participation in the study;

(2) Epithelial ovarian, primary peritoneal, or fallopian tube cancer (collectively referred to as ovarian cancer) diagnosed by histological pathology; there is no restriction on whether the patient carries a germline BRCA mutation;

(3) Patients with primary advanced epithelial ovarian cancer who have achieved clinical complete or partial remission as assessed according to RECIST v1.1 with platinum-containing chemotherapy, patients with platinum-sensitive recurrent ovarian cancer who have achieved complete or partial remission with platinum-containing chemotherapy, and patients with ovarian cancer who have achieved complete or partial remission or stable disease with multiple lines of platinum-containing chemotherapy;

(4) No prior treatment with PARP inhibitors and treatment with niraparib for at least 28 days;

Exclusion criteria: (1) ovarian cancer patients under the age of 18;

(2)Use niraparib for <28 days;

(3) Patients with ovarian cancer with histologically confirmed malignant tumors of other origins.

### Assessments

Complete response and partial response and stable disease were assessed according to RECIST1.1.

Adverse reactions were graded according to the Common Terminology Criteria for Adverse Events (CTCAE) version 5.0.

Treatment with niraparib for 28 days as a cycle.

Throughout the treatment period, It is recommended that complete blood counts in niraparib-treated ovarian cancer should be monitored weekly for the first month and monthly thereafter. In case of suspension of treatment due to Grade 3 or 4 hematological adverse events, complete blood counts should be monitored weekly after resumption of the drug until they return to normal levels (13).

Most of the adverse events in patients treated with niraparib occurred in the first 3 months, the incidence of adverse events decreased significantly after 3 months (14). Therefore, all patients in this study were followed up for more than 3 months.

The duration of follow-up was from initiation of niraparib to disease progression or permanent discontinuation or data collection cut-off.

### Dosing regimen

The retrospective RADAR analysis of the NOVA trial found that patients with a baseline bodyweight <77 kg or platelet count <150,000/microliter received an average reduced niraparib dose of 200 mg/day, without compromising treatment efficacy (15). Subsequently, the PRIMA trial modified the dosing approach to use an individualized niraparib starting dose (ISD) based on a patient’s baseline weight and platelet count base and showed an improved safety profile in 35% of patients who received an ISD compared with 65% who received a fixed starting dose (16).The initial dose of niraparib is administered on an individualized basis. The initial dose is based on basal body weight and platelet count. Patients with a basal weight ≥ 77 kg and a basal platelet count ≥ 150,000/microliter should take 300 mg/d daily, and patients with a basal weight < 77 kg and/or basal platelet count < 150,000/microliter should take 200 mg/d.

Dose reductions were allowed for drug-related adverse effects (300 to 200 mg or 100 mg; 200 mg to 100 mg) or drug interruption. For the management of adverse reactions, refer to the Chinese expert consensus on the management of adverse reactions to PARP inhibitors.

1.Generic management process for hematological adverse reactions

(1) Platelets <100.0 x 10^9/L

First occurrence: suspend niraparib for up to 28d while observing blood counts weekly until platelets return to ≥100.0 x 10^9/L. Restart treatment with the original dose of niraparib or reduce according to the protocol. If platelets are <75.0 × 10^9/L, the dose must be reduced on resumption of dosing.

Second occurrence: suspension of niraparib for up to 28d with weekly observation of blood counts until platelets return to ≥100.0 x 109/L Dose must be reduced on resumption of dosing; discontinue dosing if platelets do not return to acceptable levels within 28d of consecutive stoppages or if dosage has been reduced to the lowest possible level (100 mg/d).

(2) Neutrophils <1.0 x 10^9/L

Suspend niraparib for up to 28d while observing blood counts weekly until neutrophils return to ≥1.5 × 10^9/L. Dosage must be reduced upon resumption. Discontinue dosing if neutrophils do not return to acceptable levels within 28d of continuous discontinuation or if the dose has been reduced to the lowest possible level (100 mg/d).

(3) Hb <80g/L

Suspend niraparib for up to 28d while observing blood counts weekly until Hb returns to ≥90g/L. Dosage must be reduced upon resumption. Discontinue if Hb does not return to acceptable levels within 28 d of continuous discontinuation or if the dose has been reduced to the lowest possible level (100 mg/d).

(4) The occurrence of an adverse reaction of a lower grade than the one corresponding to the appeal should first be observed or treated symptomatically, and treatment with niraparib should be continued.

(5) Decrease in white blood cell count: the treatment is not clearly defined, and needs to be combined with the neutropenia and the patient’s symptoms and other comprehensive decisions.

(6) Definite diagnosis of myelodysplastic syndrome or acute myeloid leukemia

Permanent withdrawal.

2. Generic management process for non-hematological adverse reactions

(1) Grade 1: Continue niraparib therapy and symptomatic management as necessary.

(2) Grade 2: Continue niraparib therapy; consider interrupting therapy if adverse effects are not controlled with symptomatic or prophylactic therapy.

(3) Grade 3–4: Suspend niraparib therapy until it is reduced below Grade 1; if the adverse reaction is nausea, vomiting, or diarrhea, therapy may be continued under pharmacological control; if treatment is interrupted due to an adverse reaction, a reduction in dosage should be considered upon resumption of therapy (especially after a second interruption of dosing due to the same adverse reaction); if Grade 3/4 toxicity persists for more than 28 d after a reduction in the lowest effective therapeutic dose has been made, discontinue the niraparib therapy.

### BRCA detection

The detection technology is target region capture + high-throughput sequencing. Detection of exon coding region and exon-intron junction region +/-20bp region of BRCA1/BRCA2 gene.

### Statistical methods

SPSS 29.0 software was used for statistical analysis, frequency and percentage descriptions were used for count data, and Fisher exact test were used for correlation analysis.

## Results

### Patient characteristics

A total of 40 patients diagnosed with ovarian cancer and treated with platinum-based chemotherapy and niraparib were included in this study. The median follow-up time after starting niraparib treatment was 15.0 months (range: 2.2–32.1 months). The median age of the patients was 56 years (range: 24–75 years). There were 6 cases (15%), 31 cases (77.5%) and 3 cases (7.5%) of FIGO stage II, III and IV, respectively. Most patients had serous carcinoma (32 (80%)) and endometrioid carcinoma (4 (10%)). All the patients weighed less than 77KG and 11 of them had a platelet count less than 150*10^9/L. Twenty-eight individuals underwent BRCA testing, including 5 with BRCA1/2 mutations and 23 with BRCA wild-type. The baseline data of the patients are shown in **Table 1**.

**Table 1:** Baseline characteristics in 40 patients.

Characteristic	Number of patients (percent)	Characteristic	Number of patients (percent)
**Median age years(range)**	56(24–75)	**Front line chemotherapy cycles**	
≤59	25(62.5)	≤5	5(12.5)
>59	15(37.5)	6–9	34(85)
**Median baseline CA125(range)**	10.02 (2.15–59.20)	10	1(2.5)
**Baseline body weight**		**Clinical response after platinum-based chemotherapy**	
≥77kg	0	Complete response	36(90)
<77kg	40(100)	Partial response	2(5)
**International FIGO stage**		Stable disease	2(5)
II	6(15)	**Platelet count**	
III	31(77.5)	≥150*10^9/L	29(72.5)
IV	3(7.5)	<150*10^9/L	11(27.5)
**Presence of other comorbidities**		**BRCA status**	
Yes	38(95)	BRCA1 mutation	2(5)
No	2(5)	BRCA2 mutation	3(7.5)
**ECOG score**		BRCA wild-type	23(57.5)
0	39(97.5)	BRCA unknown	12(30)
1	1(2.5)	**Histological type**	
2	0	Serous	32(80)
**Surgical outcome**		Endometrioid	4(10)
R0	37(92.5)	Other	4(10)
R1	1(2.5)	**Prior use of bevacizumab**	
No surgical	2(5)	Yes	7(17.5)
**Type of surgery**		No	33(82.5)
NACT+IDS	21	**Niraparib time was used**	
Comprehensive staged surgery	17	<3 months	2(5)
No surgical	2	≥3 months	38(95)
**Prior lines of chemotherapy**		**Platinum type at the time of frontline chemotherapy**	
1	31(77.5)	carboplatin	34(85)
>1	9(22.5)	cis-platinum+carboplatin	3(7.5)
		Oxaliplatin+carboplatin	3(7.5)

Values are reported as frequency (n [%]) or as mean (range).

### Safety

Forty patients were included in the analysis. There were 40 cases (100%) and 30 cases (75%) of hematologic adverse reactions of any grade during platinum-based chemotherapy and niraparib treatment, including 35 cases (87.5%) and 19 cases (47.5%) of leucopenia, 37 cases (92.5%) and 23 cases (57.5%) of erythropenia, respectively. The incidences of anemia, thrombocytopenia and neutropenia were 35(87.5%) vs 22 (55%), 16(40%) vs 12 (30%), 34(85%) vs 14 (35%).The P values were 0.012, 0.065, 0.625, 0.041, 1.000 and <0.001, respectively. In the two periods, any grade of hematological adverse reactions including leukopenia, erythropenia, anemia and neutropenia were statistically significant. There were 28 cases (70%) and 16 cases (40%) with grade≥ 3 hematological adverse reactions, including 12 cases (30%) and 5 cases (12.5%) with grade ≥3 leukopenia, and 1 case (2.5%) and 3 cases (7.5%) with grade ≥3 erythropenia, respectively. Grade ≥ 3 anemia occurred in 18 cases (45%) versus 8 cases (20%), grade ≥3 thrombocytopenia in 6 cases (15%) versus 5 cases (12.5%), grade ≥3 neutropenia in 23 cases (57.5%) versus 7 cases (17.5%), P values were: 0.012, 0.065, 0.625, 0.041, 1.000, < 0.001. Grade ≥3 hematological adverse reactions including anemia and neutropenia in the two periods were statistically significant. There were 10 cases (25%) and 7 cases (17.5%) of severe hematologic toxicity (grade≥ 4), respectively (p = 0.549). The data are presented in **Table 2**.

**Table 2:** TRAE.

TRAE	PBC	Niraparib	p value
	no. of patients(%)		
Any*	40 (100)	30 (75)	0.002
Grade ≥3*	28 (70)	16 (40)	0.012
Serious*	10 (25)	7 (17.5)	0.549
Any grade white blood cell count decreased	35 (87.5)	19 (47.5)	<0.001
Grade ≥3 white blood cell count decreased	12 (30)	5 (12.5)	0.065
Any grade red blood cell count decreased	37 (92.5)	23 (57.5)	<0.001
Grade ≥3 red blood cell count decreased	1 (2.5)	3 (7.5)	0.625
Any grade anemia	35 (87.5)	22 (55)	<0.001
Grade ≥3 anemia	18 (45)	8 (20)	0.041
Any grade platelet count decreased	16 (40)	12 (30)	0.424
Grade ≥3 platelet count decreased	6 (15)	5 (12.5)	1.000
Any grade neutrophil count decreased	34 (85)	14 (35)	<0.001
Grade ≥3 neutrophil count decreased	23 (57.5)	7 (17.5)	<0.001

*: treatment-related hematologic adverse events.

During the use of platinum-based chemotherapy, 40 patients had hematologic adverse effects of any grade and 28 patients had hematologic adverse effects of grade ≥3. During niraparib maintenance therapy, adverse events of any grade occurred in 38 patients (95%), and bone marrow suppression of any grade occurred in 30 patients, of whom 16 patients had grade≥3 bone marrow suppression. There were 28 cases (70%) of non-hematologic adverse reactions, all of which were grade 1–2, and the most common adverse reactions were fatigue, nausea and Vomiting. No new safety signals were found. The data are presented in **Table 3**.

**Table 3:** Summary of adverse events.

Adverse event	niraparib maintenance therapy	
	Any grade	Grade≥ 3
	number of patients (percent)	
Nausea	14(35%)	
Vomiting	9(22.5%)	
stomachache	5(12.5%)	
Dyspepsia	2(5%)	
Decreased appetite	6(15%)	
Fatigue or asthenia	13(32.5%)	
Abdominal distention	3(7.5%)	
Constipation	7(17.5%)	
Headache	1(2.5%)	
Insomnia	5(12.5%)	
Orbital pain	2(5%)	
A foreign body sensation in the chest	1(2.5%)	
Maculopapular rash	2(5%)	
Dark skin	5(12.5%)	
loss of weight	1(2.5%)	
Elevation of blood pressure	1(2.5%)	
white blood cell count decreased	19(47.5%)	5(12.5%)
red blood cell count decreased	23(57.5%)	3(7.5%)
Thrombocytopenia	12(30%)	5(12.5%)
Neutropenia	14(35%)	7(17.5%)
Anemia	22(55%)	8(20%)
Led to dose reduction	18(45%)	
Led to discontinuation of intervention	18(45%)	
Led to dose interruption	2(5%)	

## Discussion

Platinum-based drugs inhibit tumor cell proliferation by interfering with DNA replication and transcription by binding to DNA (17, 18).The PI3K pathway is frequently upregulated in epithelial ovarian cancer and plays an important role in chemoresistance and preservation of genomic stability, as it is implicated in many processes of DNA replication and cell cycle regulation. The inhibition of the PI3K may lead to genomic instability and mitotic catastrophe through a decrease of the activity of the spindle assembly checkpoint protein Aurora kinase B and consequently increase of the occurrence of lagging chromosomes during prometaphase (19). BRCA1/2 mutations are also associated with high sensitivity for platinum groups. Patients with BRCA mutations have improved overall response to platinum-based therapy, which is associated with longer survival in patients with BRCA-mutated ovarian cancer (17, 20).PARP enzymes, especially PARP-1 and PARP-2, play a key role in the repair of DNA single-strand breaks. Inhibition of PARP leads to the accumulation of single-strand breaks, leading to the collapse of the replication strand and the accumulation of double-strand breaks, which are usually repaired by homologous recombinases. There have been six primary pathways of DNA damage repair (DDR) identified, which are variably used to address double-strand DNA breaks (DSB) and single-strand DNA breaks damage from a variety of mechanisms of injury. Homologous recombination (HR) and nonhomologous end joining (NHEJ) recombination are the two major pathways responsible for repairing DSB (21). HR pathways become active in the S/G2 phase due to the availability of a sister chromatid, whereas NHEJ repairs DSB throughout all cell cycle phases except the M phase. NHEJ is faster than HR and mainly occurs in the G1 phase, Beyond the already-known proteins, such as Ku70/80, DNA-PKcs, Artemis, DNA pol λ/μ, DNA ligase IV-XRCC4, and XLF, new proteins are involved in the NHEJ, namely PAXX, MRI/CYREN, TARDBP of TDP-43, IFFO1, ERCC6L2, and RNase H2 (22, 23). Among them, MRI/CYREN has dual role, as it stimulates NHEJ in the G1 phase of the cell cycle, while it inhibits the pathway in the S and G2 phases (24). Ovarian cancers with BRCA1/BRCA2 mutations or other HRDs are particularly sensitive to PARP inhibitors because the accumulation of unrepaired DNA breaks leads to cell death (25, 26).This is known as “synthetic lethality”。Niraparib is a highly selective inhibitor of PARP1/2 (a nuclear protein that detects DNA damage and promotes its repair) (27), and the most common adverse effect of niraparib is myelosuppression, with most interruptions of niraparib treatment due to myelosuppressive events (16).

The anti-tumor mechanism of PARP inhibitors (PARPi) overlaps with platinum-based drugs in DNA damage repair pathways. Patients who are effective to platinum-based chemotherapy are also more likely to be sensitive to PARPi. The dose-limiting toxicity of carboplatin is myelosuppression, and its non-hematologic adverse reactions are milder and fewer than those of cisplatin (28–30), and several studies have shown that the most common ≥grade 3 adverse reactions of niraparib are also hematologic adverse reactions (31–33). In this real-world study, we observed a lower rate of hematologic adverse effects with niraparib than with platinum-based chemotherapy in patients with advanced ovarian cancer. Niraparib maintenance therapy is better tolerated than platinum-based chemotherapy in this study. Due to the small sample size, larger sample size is needed for further verification.

In this study, all patients received a starting dose of niraparib of 200mg/d according to their basal body weight and basal platelet count, which was consistent with the Chinese prospective study (31, 32). The most common adverse reactions of any grade were hematologic adverse reactions, nausea, and fatigue, and there were 16 cases of ≥ grade 3 adverse reactions, all of which were hematologic adverse reactions, which were similar to the results of the NORA (31)study. A meta-analysis showed that niraparib adverse effects were significantly dose-related, and most of them could be controlled by suspending therapy, reducing dose, and treating symptomatic therapy (34).In this study, during the maintenance treatment with niraparib, 16 patients experienced grade ≥3 adverse reactions, 18 patients reduced their dose due to adverse drug reactions, 18 patients discontinued their medication due to adverse drug reactions, 1 patient spontaneously terminated the drug due to stomach pain after taking the drug, and 1 patient terminated the drug due to recurrent ≥ grade 3 bone marrow suppression, which is consistent with the results of the meta-analysis (35)of the current clinical trial. In the context of the new crown epidemic, 8 patients stopped taking the drug for 1–4 weeks due to new coronavirus infection, and all patients passed the new coronavirus infection period safely.

Niraparib has a long treatment cycle and is therefore particularly important for the management of adverse effects. Standardized whole-process management, including pre-medication evaluation and adequate doctor-patient communication, standardized detection during medication and timely treatment of AEs, can reduce and reduce the occurrence of AEs, increase the safety of medication, and improve the compliance of patients, so as to further ensure the efficacy of niraparib treatment cycle is long, so the management of adverse reactions is particularly important. Standardized whole-process management, including pre-medication evaluation and adequate doctor-patient communication, standardized detection during medication and timely treatment of AEs, can reduce and reduce the occurrence of AEs, increase the safety of medication, and improve the compliance of patients, so as to further ensure the efficacy. Myelosuppression is a dose-limiting toxicity of most platinum drugs and niraparib (17, 30).This study evaluates whether there are treatment-related hematological adverse reactions between platinum-based chemotherapy and niraparib treatment, and provides a clinical reference for the whole process of precise and standardized management of ovarian cancer patients.

This study shows that any grade of adverse blood reactions, including (decreased white blood cells, decreased red blood cells, anemia, and neutrophils), occurred during platinum-based chemotherapy and niraparib maintenance therapy in patients with ovarian cancer, and there was a correlation between grade ≥ grade 3 adverse reactions including (anemia, neutrophil decline). There was no statistically significant correlation between any grade of anemia and grade ≥ grade 3 leukocytopenia, grade ≥ grade 3 erythrocyte decline, and grade 3 thrombocytopenia ≥ the two periods. Due to the small sample size, it is not possible to obtain a valid correlation strength analysis, which requires more data for further validation. Based on this study, it is believed that the occurrence of serious hematologic adverse reactions with platinum-based chemotherapy may be a risk factor for patients to develop serious hematologic adverse reactions in maintenance therapy with niraparib. Based on the results of this study, the use of niraparib treatment should take into account whether the patient has experienced ≥ grade 3 hematological adverse reactions, especially anemia and ≥ grade 3 neutropenia during the first-line platinum-based chemotherapy, and the timing of drug administration can be determined according to the patient’s condition. More attention should be paid to the monitoring and management of hematologic toxicity in patients with a history of ≥ grade 3 hematologic toxicity during the subsequent treatment with niraparib. Doctors can strengthen the relevant medical education for these patients and inform patients to see a doctor in time when they have symptoms related to blood adverse reactions such as pale complexion, fatigue, fever, gingival bleeding, and skin ecchymosis. Increase the frequency of hematological analysis and testing for this group of patients, as appropriate, and intervene as early as possible to standardize treatment in the event of adverse hematological reactions in patients. In order to better guide the clinic, accumulate clinical medication experience, increase patient compliance, so that patients can better benefit from PARP inhibitors.

## Conclusion

1. In this real-world practice, we observed that patients with advanced ovarian cancer who experienced any grade and grade ≥3 TRAE during chemotherapy were well tolerated when treated with niraparib, particularly the incidence of any grade and grade ≥3 anemia, and neutrophil count decreased during niraparib treatment were significantly lower compared with that during chemotherapy.

2. For patients with ovarian cancer who have experienced grade ≥3 hematological adverse reactions during prior platinum-based chemotherapy, greater attention should be paid to the monitoring and management of hematological adverse reactions during subsequent treatment with niraparib.

## Data availability statement

The datasets presented in this study can be found in online repositories. The names of the repository/repositories and accession number(s) can be found in the article/supplementary material.

## Ethics statement

The studies involving humans were approved by Ethics Committee of First Affiliated Hospital of Gannan Medical University. The studies were conducted in accordance with the local legislation and institutional requirements. The participants provided their written informed consent to participate in this study. Written informed consent was obtained from the individual(s) for the publication of any potentially identifiable images or data included in this article.

## Author contributions

LW: Data curation, Investigation, Writing – original draft, Software. JZ: Project administration, Supervision, Writing – review & editing, Data curation. HW: Investigation, Writing – original draft. WH: Supervision, Writing – review & editing. CF: Writing – original draft.
